# Characteristics of Microbial Distribution in Different Oral Niches of Oral Squamous Cell Carcinoma

**DOI:** 10.3389/fcimb.2022.905653

**Published:** 2022-08-15

**Authors:** Fujiao Nie, Lihua Wang, Yingying Huang, Pishan Yang, Pizhang Gong, Qiang Feng, Chengzhe Yang

**Affiliations:** ^1^ Department of Periodontology, School and Hospital of Stomatology, Cheeloo College of Medicine, Shandong University, Jinan, China; ^2^ Shandong Key Laboratory of Oral Tissue Regeneration & Shandong Engineering Laboratory for Dental Materials and Oral Tissue Regeneration, Jinan, China; ^3^ Department of Human Microbiome, School and Hospital of Stomatology, Cheeloo College of Medicine, Shandong University, Jinan, China; ^4^ Department of Oral and Maxillofacial Surgery, Qilu Hospital of Shandong University, Jinan, China; ^5^ Institute of Stomatology, Shandong University, Jinan, China

**Keywords:** oral squamous cell carcinoma, oral microbiome, distribution characteristics, oral niches, clinicopathological features

## Abstract

Oral squamous cell carcinoma (OSCC), one of the most common malignant tumors of the head and neck, is closely associated with the presence of oral microbes. However, the microbiomes of different oral niches in OSCC patients and their association with OSCC have not been adequately characterized. In this study, 305 samples were collected from 65 OSCC patients, including tumor tissue, adjacent normal tissue (paracancerous tissue), cancer surface tissue, anatomically matched contralateral normal mucosa, saliva, and tongue coat. 16S ribosomal DNA (16S rDNA) sequencing was used to compare the microbial composition, distribution, and co-occurrence network of different oral niches. The association between the microbiome and the clinical features of OSCC was also characterized. The oral microbiome of OSCC patients showed a regular ecological distribution. Tumor and paracancerous tissues were more microbially diverse than other oral niches. Cancer surface, contralateral normal mucosa, saliva, and tongue coat showed similar microbial compositions, especially the contralateral normal mucosa and saliva. Periodontitis-associated bacteria of the genera *Fusobacterium*, *Prevotella*, *Porphyromonas*, *Campylobacter*, and *Aggregatibacter*, and anaerobic bacteria were enriched in tumor samples. The microbiome was highly correlated with tumor clinicopathological features, with several genera (*Lautropia*, *Asteroleplasma*, *Parvimonas*, *Peptostreptococcus*, *Pyramidobacter*, *Roseburia*, and *Propionibacterium*) demonstrating a relatively high diagnostic power for OSCC metastasis, potentially providing an indicator for the development of OSCC.

## Introduction

Oral squamous cell carcinoma (OSCC), one of the most common malignant tumors of the head and neck, accounting for approximately 90% of oral cancers (Chi et al., 2015), is characterized by invasiveness, rapid development, early metastasis, and poor prognosis (Siegel et al., 2020). The incidence of OSCC has increased significantly in recent years, accounting for over 370,000 new cases and 170,000 deaths worldwide in 2020, while the five-year survival rate remained between 50% and 60% (Choi et al., 2014; Patel et al., 2016; van Dijk et al., 2016). The pathogenesis of OSCC is multifactorial, among which tobacco, alcohol, chewing betel nut and human papillomavirus (HPV) have been demonstrated as risk factors (Chi et al., 2015). Recently, chronic pathogenic infections, including Candida infection and periodontitis, have been recognized as high-risk factors for the occurrence and development of OSCC (Peres et al., 2019).

More than 700 bacterial species have been detected in the oral cavity, and the oral microbiome, which is considered highly diverse compared to other body sites, encompasses a wide variety of microorganisms from different niches, such as tongue, buccal mucosa, and saliva, reflecting site-specificity in oral species (Minarovits, 2021). Human oral microbiome composition are closely correlated with oral and systematic health (Verma et al., 2018). For example, the oral pathogen, *Fusobacterium nucleatum*, has been well studied as an oncogenic pathogen of colorectal cancer (Brennan and Garrett, 2019). Additionally, more and more oral pathogenic microbes, such as *Porphyromonas gingivalis*, *Actinobacillus actinomycetemcomitans*, *Tannerella forsythus*, and *Prevotella intermedius*, have been demonstrated to be risk factors for esophageal, gastric, and pancreatic carcinomas (Mascitti et al., 2019).

Dysbiosis of the oral microbiome has gained significant attention as a potential oncogenic factor of OSCC in recent years. Most bacterial taxa isolated from tumor tissue were periodontitis-related and saccharolytic or aciduric species in comparison to non-tumorous paracancerous mucosal tissue (Hooper et al., 2007; Zhao et al., 2017), while the abundance of *Streptococcus* progressively decreased with the progression of OSCC (Chattopadhyay et al., 2019; Ganly et al., 2019). Oral inflammatory-related bacteria (e.g., *Fusobacterium nucleatum* and *Pseudomonas aeruginosa*) are more likely to be enriched in OSCC tissue (Al-Hebshi et al., 2017b; Perera et al., 2018). Abundances of the bacteria *Porphyromonas endodontalis* and *Peptostreptococcus anaerobius* in salivary were reported to be significantly correlated with increases of inflammatory cytokines IL-6, IL-8, TNF-α, IFN-γ, and GM-CSF in OSCC patients (Rai et al., 2021).

Studies have increasingly shown that oral microorganisms are closely related to oral cancer, while the microbial compositions in different oral niches differ significantly due to the different microenvironments. However, in OSCC patients, whether there exists a substantial difference in the oral microbiome signature among intratumor and oral other niches remains scarcely reported. In this study, microbial samples of six oral niches were collected from OSCC patients. Phenotypic and functional characteristics of the oral microbiome were identified, and the association between the oral microbiome and the clinical biological behavior of OSCC was investigated.

## Materials and Methods

### Participant Recruitment and Sample Collection

The study was approved by the Ethics Committee of Qilu Hospital of Shandong University (KYLL-2017-256), and conducted in accordance with the Declaration of Helsinki. All subjects gave written informed consent before participating in the study. The patients diagnosed with OSCC for the first time based on clinical symptoms and histopathological detection were eligible for this study. Prior to sampling, no patients received any treatments such as surgery, immune therapy, radio- or chemotherapy. Exclusion criteria were the use of antibiotics in the past 3 months or diseases/conditions known to modify oral microbial composition such as pregnancy, nursing, and oral mucosal diseases. Subjects with a history of any previous cancer diagnosis or any other severe systemic disorder, such as diabetes, infectious disease, HBV, syphilis, and HIV infection, autoimmune diseases, gastrointestinal and respiratory diseases were excluded from the study.

A total of 65 patients with OSCC were recruited from the Qilu Hospital of Shandong University (Shandong, China) from 2018 to 2020. Samples from six oral niches (tumor tissue, paracancerous tissue, cancer surface, anatomically matched contralateral normal mucosa, saliva, and tongue coat) were collected according to the protocols of Human Microbiome Project (HMP) (McInnes and Cutting, 2010). Participants were instructed not to eat, drink, smoke, or to brush their teeth for at least 2 hours before sample collection. For the saliva samples, participants were asked to stop swallowing for 1 minute and to spit saliva into a 50 mL collection tube, repeating the procedure several times and finally collecting approximately 5mL saliva. The samples from the cancer surface, contralateral normal mucosa, and tongue coat niches were obtained with a sterile swab by swabbing the surface of the soft tissue with pressure for 10 times in one direction, then turning the swab 180° and swabing 10 times with the opposite side. The central 1 cm^2^ area of tongue dorsum, the entire buccal mucosa, and the entire cancer surface were samplings, avoiding the teeth and the internal tumor. Tumor tissue and paracancerous tissue samples were collected during the surgical resection of OSCC from the regions of the tumor lesion and from the adjoining clinically uninvolved normal tissue (paracancerous niche). Concisely, around 5 mm^3^ tumor samples were excised from the deep tissue of tumor mass without involving the margin, and paired normal tissues of similar size were excised from the paracancerous region 2 cm away from the edge of tumor lesions. All operation maintained consistency in the sampling process to facilitate the repetition of experimental results.

All the samples were placed in prepared oral swab preservation solution (Tris, EDTA, and antiseptic) to prevent DNA degradation and transported to the laboratory on ice within 20 mins of collection before being stored at −80°C until processing.

### DNA Extraction

DNA was extracted from all samples using DNeasy Blood and Tissue Kit (Qiagen, Hilden, Germany) following the manufacturer’s protocol. The concentration and purity of the isolated DNA were detected using 1% agarose gels electrophoresis. DNA was diluted to 1ng/μl using sterile water.

### 16S rDNA Amplification and Sequencing

Genome DNA from each sample was used as an amplification template. PCR targeting of the V3-V4 region of the 16S rDNA hypervariable region was conducted, using the primers 341F (5′-CCTAYGGGRBGCASCAG-3′) and 806R (5′-GGACTACNNGGGTATCTAAT -3′). PCR was performed in a total volume of 30μL reactions, composing 15μL of Phusion^®^ High-Fidelity PCR Master Mix (New England Biolabs), 0.2μM of forward and reverse primers, and 10 ng of the template DNA. Thermal cycling was initially used to denature samples at 95°C for 10 min, followed by 30 cycles of denaturation at 95°C for 30s, then annealing at 50°C for 30s and extension at 72°C for 40s, followed finally by elongation at 72°C for 7 min.

PCR products were then purified using a 2% agarose gel electrophoresis and GeneJET Gel Extraction Kit (Thermo Scientific, USA), selecting samples with a single amplification product for further analysis. The library was sequenced on an Illumina Hiseq 2500 platform (Novogene, Beijing, China).

### Sequencing Data Analysis

The microbiome bioinformatics platform QIIME2 was used to analyze the raw sequence. Usearch software (Usearch, version 11.0.667_i86linux64) was used to identify the Operational Taxonomic Units OTUs (Edgar, 2010). Sequence data were clustered into OTUs at 97% similarity by Usearch, and then taxonomic annotation of the representative sequences of OTUs was performed by the database Ribosomal Database Project (RDP) at a threshold of 0.8.

The alpha-diversity index reflected the species richness, evenness, and diversity in every group, and the beta-diversity was used to compare the degree of similarity or dissimilarity among different groups, both performed using the QIMME2 (Bolyen et al., 2019). Alpha-diversity was analyzed by comparing the Chao1 index, Faith-PD, observed OTUs, Shannon index, and Simpson diversity index. Principle Coordinate Analysis (PCoA) based on unweighted UniFrac were performed to visualize beta-diversity. ADONIS analysis was used to evaluate any statistical differences. Microbiome variations in different niches were analyzed at the phylum, genus, and species levels. The taxonomic composition and average relative abundance of microbiome were displayed using a histogram (R package: “ggplot2”, version 3.3.5), and the differential microbiomes between groups at the genus and species levels were demonstrated using a heatmap (R package, pheatmap, version 1.0.12).

By following the law of power-law distribution, the SparCC network was constructed using the FastSpar to analyze the correlation and interaction between flora species, as well as to identify the key flora and related flora according to the co-occurrence network of flora-species interaction (Friedman and Alm, 2012; Watts et al., 2019).

To characterize the relationship between oral microbiome and the clinical characteristics of OSCC, tumor tissue samples were divided by tumor size, grade, stage, and metastasis. The bacteria related to the clinical indicators were screened using Maaslin2 (R, version 1.6.0) at a significance threshold of p < 0.05. Subsequently, the Boruta algorithm (R package version 7.0.0) was used to select the taxa exhibiting predictive power. Finally, a random forest algorithm (R package, randomForest, version 4.6-14) was performed with 500 classification trees based on leave one out cross validation (LOOCV) to establish classification models for the diagnosis of OSCC (Basu et al., 2018). The predictive performance of each classification model was assessed using the ROC curve (R package: pROC, version 1.16.2), and for each the area under the curve (AUC) was calculated.

The functional prediction of the microbial community was investigated using a phenotypic classification based on BugBase, and metabolic pathway prediction based on Tax4Fun2 (R package version 1.1.5). OTU abundance table and representative OTU sequences were aligned against the Ref99NR database using Tax4Fun2, then Kyoto Encyclopedia of Genes and Genomes (KEGG) functional classification between samples were realized by STAMP software (version 2.1.3) (Kanehisa and Goto, 2000).

## Results

### Sociodemographic Characteristics and Statistics of the Sequencing Data

A total of 65 subjects with OSCC were recruited and 305 samples were collected from 6 different oral niches. Clinical characteristics of the study subjects were presented in the **Table 1**. 16S rDNA regions V3–V4 were amplified and sequenced successfully from each sample according to the standard process described in the Methods section. After post-processing and quality filtering, 23,451,170 reads were obtained across all samples, with 76,889 reads per sample on average. OTU detection based on the RDP database showed 2,398, 2,542, 2,030, 1,562, 1,279, 1,149 bacterial OTUs were obtained from the six niches (tumor tissue, paracancerous tissue, cancer surface, contralateral normal mucosa, saliva, and tongue coat), respectively. The rarefaction curve showed that all samples reached saturation, indicating that almost all detectable microbial species in each sample were identified.

**Table 1:** Clinicopathological characteristics of the patients.

Variable	C (n=37)	PC (n=37)	CS (n=64)	N (n=38)	S (n=65)	T (n=64)
**Age** (mean ± SD)	57.9 ± 9.6	58.1 ± 9.7	59.5 ± 10.9	58.2 ± 10.3	59.5 ± 10.8	59.5 ± 10.9
**Males**: No. (%)	27 (73.0)	27 (73.0)	41 (64.1)	25 (65.8)	41 (63.1)	41 (64.1)
**Cancer site**:						
Tongue	15	14	23	17	24	23
Buccal	2	2	8	4	8	8
Floor of Mouth	7	7	10	5	10	10
Pharynx	1	1	1	1	1	1
Palate	3	3	6	4	6	6
Maxilla	2	2	4	2	4	4
gingiva	7	8	12	5	12	12

### Alpha and Beta Diversity Across Oral Niches

**Figure 1:**
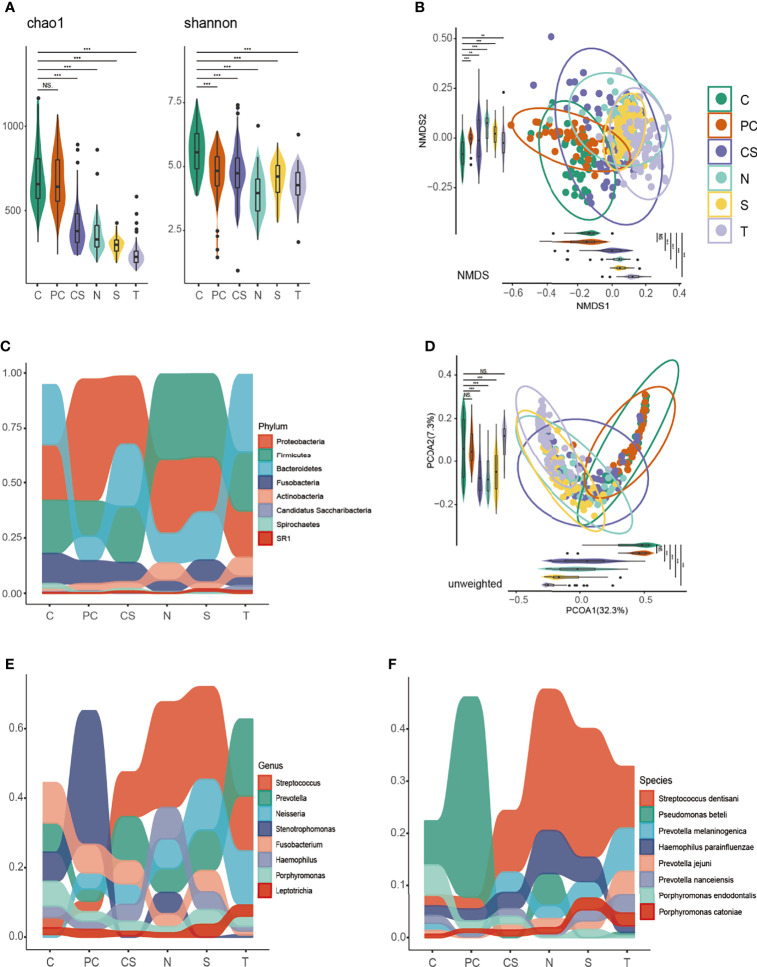
Diversity analysis and taxonomy distribution of the oral microbiome among six niches. Bacterial richness, evenness and diversity were evaluated by **(A)** Chao 1 and Shannon index. Clusters formed by **(B)** non-metric multidimensional scaling (NMDS) and **(D)** principal coordinate analysis (PCoA) based on unweighted UniFrac. Differential taxonomy distribution at the **(C)** phylum, **(E)** genus and **(F)** species level. C, tumor tissue; PC, paracancerous tissue; CS, cancer surface; N, anatomically matched contralateral normal mucosa; S, saliva; T, tongue coat. ns, no significance; **P < 0.005, ***P < 0.001.

Alpha diversity was assessed to compare the richness, uniformity, and diversity of the microbiome. Using Chao1, Faith PD, and OTU analyses (**Figure 1A** and **Supplementary Figures 1A–C**), we identified a gross spatial trend of microbial communities with species richness being highest in the tumor and paracancerous samples, while cancer surface, contralateral normal tissue, saliva, and tongue coat samples exhibited a trend of progressively decreasing richness. The Shannon and Simpson indices suggested that the diversity and uniformity of bacteria were significantly higher in the tumor tissue than in other niches (*P* < 0.001).

Non-metric multidimensional scaling (NMDS) and principal coordinate analysis (PCoA) were conducted to measure the niche-related differences in microbial communities and their clustering relationships. The PCoA was based on unweighted UniFrac. As shown in **Figures 1B, D**, the six niches formed separate clusters, and this difference was found using ADONIS to be statistically significant. Further ADONIS analysis of the distributions of samples from different niches showed that the core regions of tumor samples and paracancerous samples were highly similar (*P* > 0.05), suggesting similar microbial diversities in those two niches, while the contralateral normal mucosa, saliva, and tongue coat were different from the tumor and paracancerous samples, but similar to each other.

### Generality and Discrepancy of Microbial Composition Across Oral Niches

The generality and discrepancy of oral microbial composition was compared across niches to elucidate the microbial transitions along different oral niches. As indicated in **Figures 1C, E, F**, there were significant differences in oral microbial composition across niches. *Proteobacteria*, *Firmicutes*, *Bacteroidetes*, *Fusobacteria*, and *Actinobacteria* were the predominant microorganisms in all niches at the phylum level. Among these, *Proteobacteria*, *Firmicutes*, and *Bacteroidetes* accounted for the greatest proportion. The microbiome of contralateral normal mucosa and saliva samples showed great similarity at the phylum, genus, and species levels. Further, *Pseudomonas beteli* was the most abundant species in both tumor and paracancerous tissue samples, whereas, in the other four niches, *Streptococcus dentisani* accounted for the largest proportion, indicating a more similar community composition between tumor and paracancerous tissues, while the other four niches were more similar to each other. We propose that it is the fluidity of saliva and activity of tongue that caused microbial composition of this saliva, tongue coat, cancer surface, and contralateral normal mucosa resemble each other, especially contralateral normal mucosa and saliva (Mager et al., 2003; Li et al., 2020). This finding might facilitate selecting sampling sites for microbial composition research.

### Microbiome Profiles of Tumor and Paracancerous Tissues

The tumor and paracancerous tissues were sampled from the surgically resected tumor and adjacent non-tumorous tissues, while the other four groups were sampled from liquid saliva and using swabs. This may be a major contributor to the microbial differences observed. Thus, subsequent analyses focused on the variations between tumor and paracancerous tissue samples to characterize the dysbiosis of the oral microbiome associated with OSCC.

**Figure 2:**
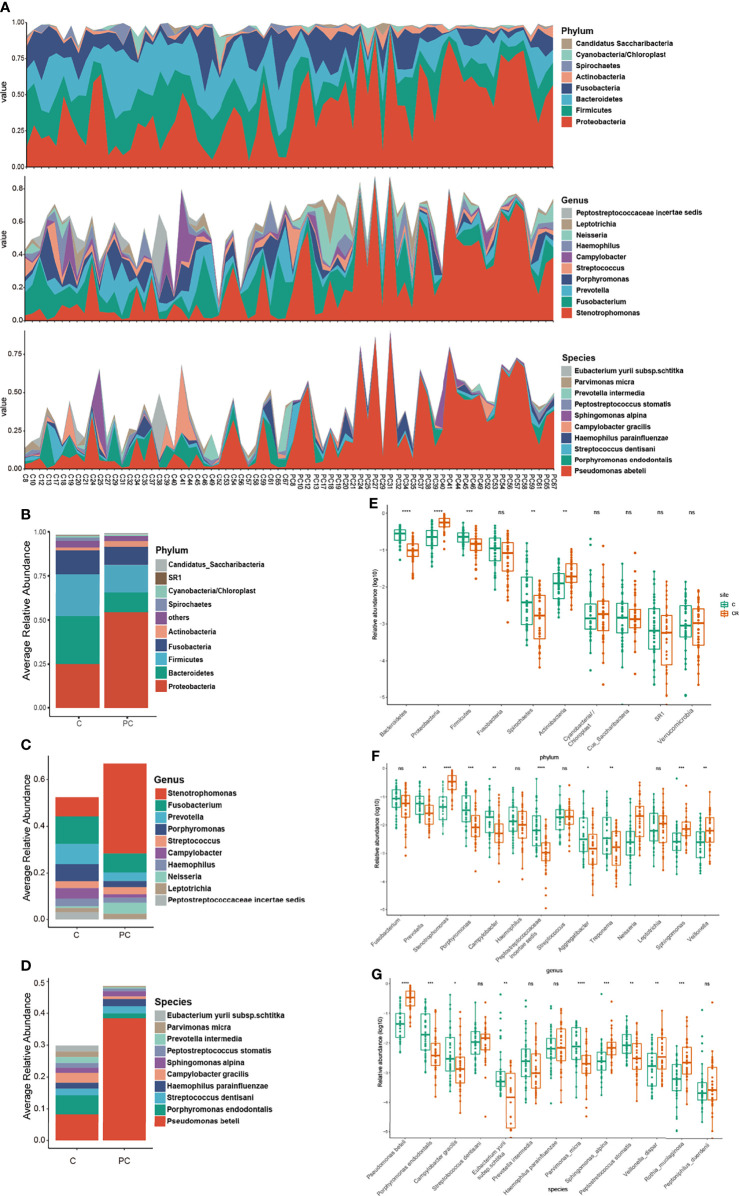
**(A)** The comparison of taxonomic composition between tumor and paracancerous tissue samples. Distribution of relative abundance of microbiome in the tumor tissue and paracancerous tissue of each OSCC subject at the phylum, genus, and species level. Histograms described relative abundances of the **(B)** top 10 phylum, **(C)** 10 genera, and **(D)** top 10 species in the tumor tissue and paracancerous tissue. **(E–G)** Prevalence of bacterial phylum, genera and species associated with tumor and paracancerous tissue of OSCC subjects. C, tumor tissue; PC, paracancerous tissue. ns, no significance; *P *<* 0.05, **P < 0.005, ***P < 0.001, ****P < 0.0001.

The bacterial composition of tumor tissue was noticeably different in comparison to the paracancerous tissue in most patients, suggesting a shift in bacterial colonization. The results of phylum-level taxonomic analysis in **Figures 2A, B, E** show that *Proteobacteria* were overwhelmingly dominant in paracancerous tissue, while significantly reduced in tumor tissue, while *Firmicutes*, *Bacteroidetes*, *Fusobacteria*, and *Spirochaetes* were increased in tumor tissue. At the genus level, tumor tissue showed a greater abundance of *Fusobacterium*, *Prevotella*, *Porphyromonas*, *Campylobacter*, *Aggregatibacter*, *Treponema*, and *Peptostreptococcus*, and a lower prevalence of *Stenotrophomonas* (the most abundant in control tissue), *Neisseria*, *Sphingomonas*, and *Veillonella* (**Figures 2A, C, F**, *P* < 0.05). At the species level, *Pseudomonas beteli* accounted for the highest proportion in paracancerous tissue but was significantly reduced in tumor tissue. *Rothia mucilaginosa*, *Sphingomonas alpina*, and *Veillonella dispar* were also significantly decreased in tumor tissue. Conversely, *Porphyromonas endodontalis*, *Campylobacter gracilis*, *Peptostreptococcus stomatis*, *prevotella intermedia*, *Eubacterium vuril subsp schtika*, and *Parvimonas micra* were significantly increased in tumor tissue (**Figures 2D, G**, *P* < 0.05).

**Figure 3:**
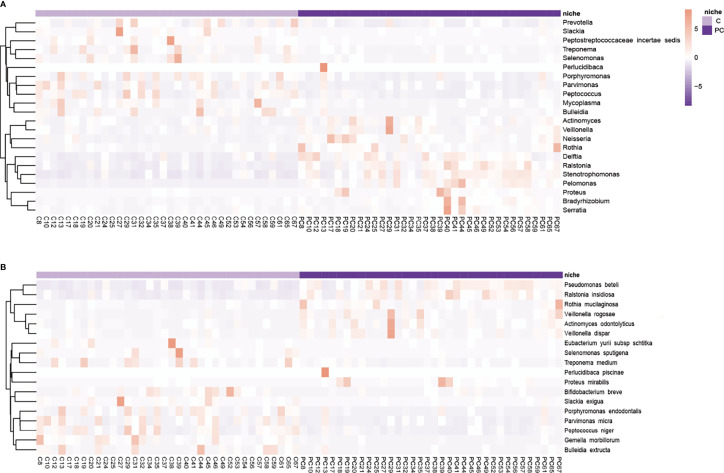
Hierarchically clustered heatmap analysis of differential microflora. The **(A)** genera and **(B)** species level microbial community indicated the formation of two main clusters, separating tumor tissue and paracancerous tissue of each subject. Color key of heat map was shown on the right side of the figure. It shows microbes with high abundance in red, microbes with low abundance in purple. C, tumor tissue; PC, paracancerous tissue.

To further distinguish differences of the microbial community between cancer tissue and paracancerous tissue, differential clusters were visualized using a heatmap (see **Figure 3**). 22 genera were identified as being differentially enriched between tumor and paracancerous tissue samples. Compared to paracancerous tissue, *prevotella*, *Slackia*, *Peptostreptococcus*, *Treponema*, *Selenomonas*, *Porphyromonas*, *parvimonas*, *Peptococcus*, *Mycoplasma*, and *Bulleidia* were enriched in tumor tissue. Interestingly, these are all anaerobic or facultative anaerobes. Conversely, Actinomyces, Veillonella, Neisseria, Rothia, Delftia, Ralstonia, Stenotrophomonas, pelomonas, Proteus, Bradyrhizobium, and Serratia, were less abundant in tumor tissue. There were also differences in the species level cluster distributions, whereby tumor tissue exhibited increased *Porphyromonas endodontalis* and *Parvimonas micra*, and reduced *Pseudomonas beteli*, *Rothia mucilaginosa*, *Sphingomonas alpina*, and *Veillonella rogosae*.

### SparCC Analysis of the Co-Occurrence Network and Core Microbiome

**Figure 4:**
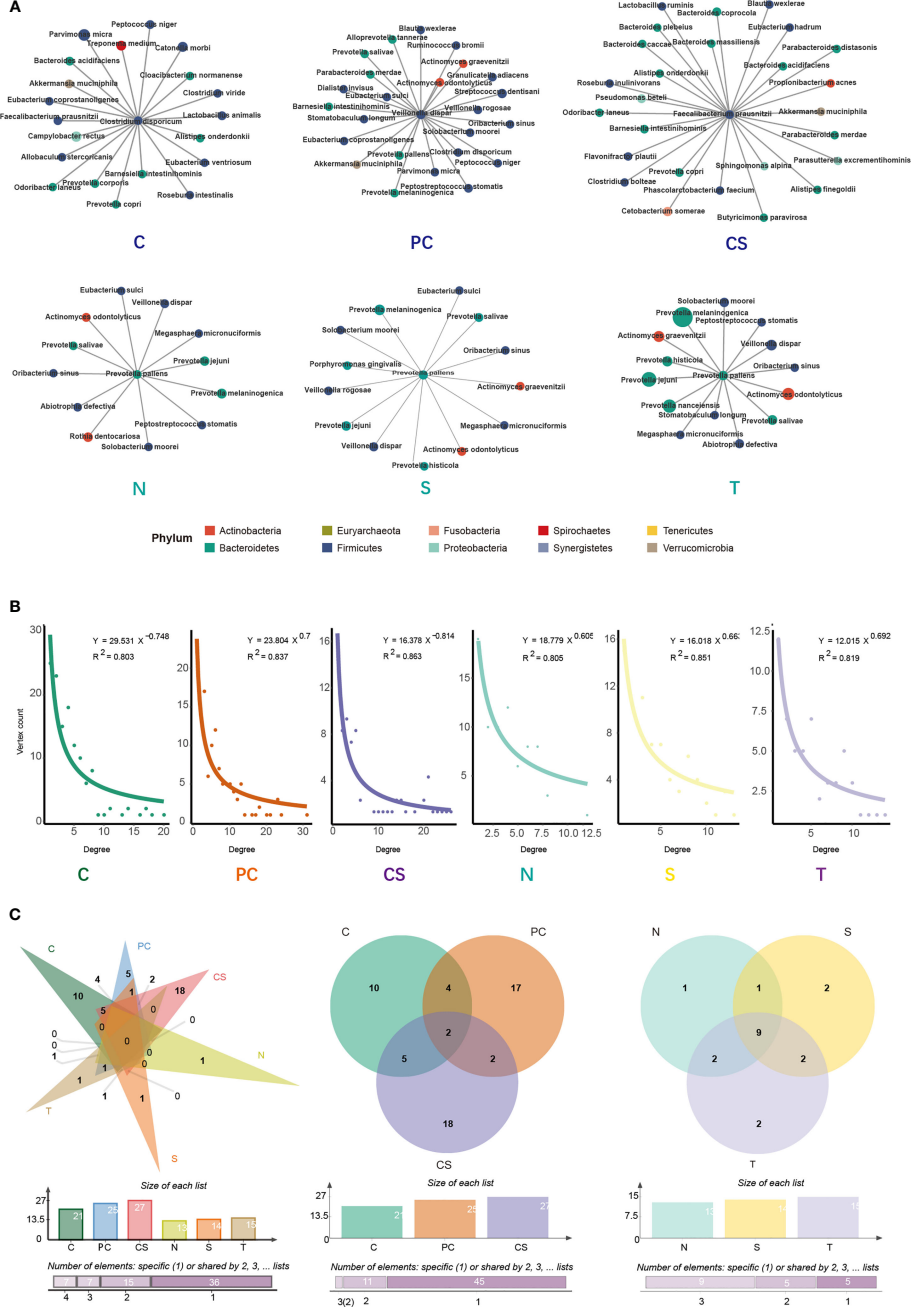
SparCC analysis of the co-occurrence network and core microbiome. **(A)** Key species network in tumor tissue, paracancerous tissue, cancer surface, anatomically matched contralateral normal mucosa, saliva, and tongue coat. Each node represents a specie, and each edge represents a significant co-occurrence relationship. **(B)** SparCC network followed the law of power-law distribution. **(C)** Venn diagram depicts the overlap of bacterial species among the different niches. C, tumor tissue; PC, paracancerous tissue; CS, cancer surface; N, anatomically matched contralateral normal mucosa; S, saliva; T, tongue coat.

To describe the microbial symbiosis and the connections across different bacterial communities, the microbial co-occurrence network was constructed using SparCC analysis (**Supplementary Figure 2**). SparCC provides a novel method of sequencing data to infer correlations between species (Rai et al., 2021). The degree of microbial symbiosis in the network corresponded with the power-law distribution, indicating the ecologic characteristics of the oral microbiome in OSCC patients (**Figure 4B**). The nodes of the network represented the bacterial species, while the edges between nodes represent ecological relationships between species. The node with most edges was considered as the key species. As shown in **Supplementary Figure 2**, tumor and paracancerous group were the two densest clusters among the six niches, indicating increased network complexity of the two groups. *Clostridium disporicum* and *Veillonella dispar*, with the most associations in the clusters, were the key species in tumor and paracancerous tissue samples, respectively (**Figure 4A**). On the cancer surface, the key species was *Faecalibacterium prausnitzi*, one of the most abundant and important commensal bacteria in the human intestinal microbiome. Contralateral normal mucosa, saliva, and tongue coat samples exhibited the same key species, *Prevotella pallens*, indicating that this species heavily participates in the bacterial ecology structure of the 3 niches, and the three niches presented relatively similar microbial community compositions.

As showcased in **Figure 4C**, the Venn diagram was generated by the bacterial species in **Figure 4A**, and it represented the overlap of species among different groups. For the species directly connected to the key species, there were no overlapping species across all six groups. Besides, 2 species overlap across the tumor tissue, paracancerous tissue, and cancer surface, and 9 species overlap among the other three groups were observed, suggesting that the controlateral normal mucosa, saliva, and tongue coat had similar microbial structure in their co-occurrence networks. These findings were consistent with the results shown above.

### Diagnostic Performance of the Oral Microbiome in Discriminating OSCC

**Figure 5:**
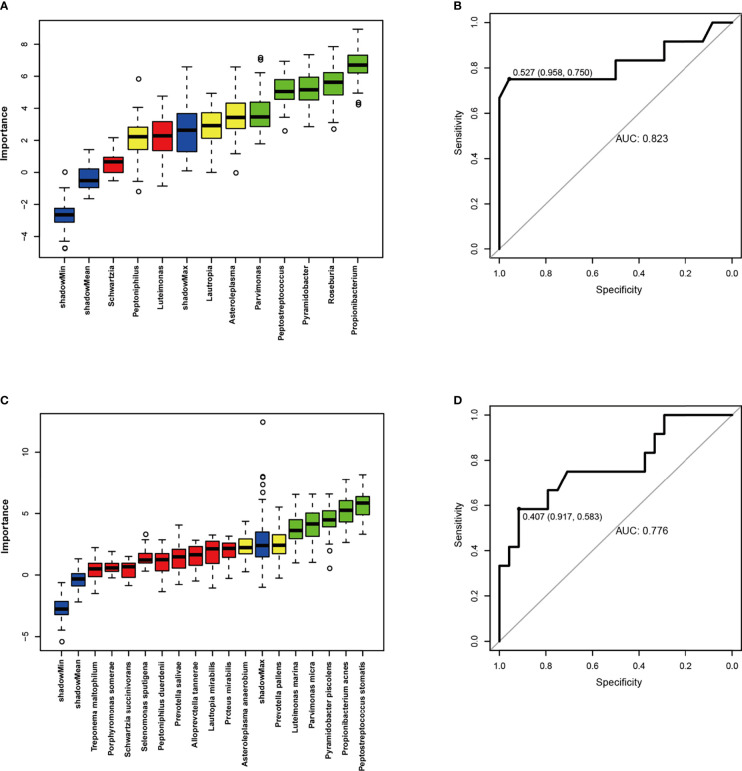
ROC curve analyses were performed to evaluate the diagnostic performance of oral microbiome for OSCC metastasis. **(A, C)** Boruta algorithm was used for feature selection and to assess the importance of variables. **(B, D)** ROC curves were constructed to predict diagnostic power of microbiome for tumor metastasis. It suggested that higher abundance of Lautropia, Asteroleplasma, Parvimonas, Peptostreptococcus, Pyramidobacter, Roseburia and Propionibacterium in the tumor tissue appeared to be associated with higher rates of tumor metastases.

To assess the association between the microbial community and the clinical index of OSCC, tumor samples were grouped by tumor size (1: T1, 2: T2, 3: T3, 4: T4), grade (1: highly differentiated, 2: moderately differentiated, 3: poorly differentiated), stage (1: T1 N0 M0, 2: T2 N0 M0, 3: T1-2 N1 M0 and T3 N0-1 M0, 4: T1-3 N2 M0 and T4a N0-2 M0), and metastasis (1: metastasis, 2: no metastasis) using the TNM classification of the American Joint Committee on Cancer (AJCC Cancer Staging Manual, Eighth Edition). MaAsLin2 analysis was conducted to identify bacterial differences between groups. ROC curve analysis was performed to evaluate whether the relative abundances of specific microorganisms reflected any clinical characteristics. As presented in **Figures 5A, B**, the ROC curve was constructed to assess the diagnostic ability of selected bacterials for OSCC metastasis. The AUC reached 0.823 at the genus level, indicating a good level of diagnostic performance of the genera *Lautropia*, *Asteroleplasma*, *Parvimonas*, *Peptostreptococcus*, *Pyramidobacter*, *Roseburia*, and *Propionibacterium* to predict tumor metastasis. The species *Parvimonas micra*, *Prevotella pallens*, *Propionibacterium acnes*, *Pyramidobacter piscolens*, *Luteimonas marina*, and *Peptostreptococcus stomatis* also correlated significantly with metastasis (AUC = 0.776; **Figures 5C, D**). However, the associations between bacterial populations and other clinicopathological characteristics (tumor size, grade, and stage) were poor (**Supplementary Figure 3**).

### Prediction of Microbiome Phenotype and Functions

**Figure 6:**
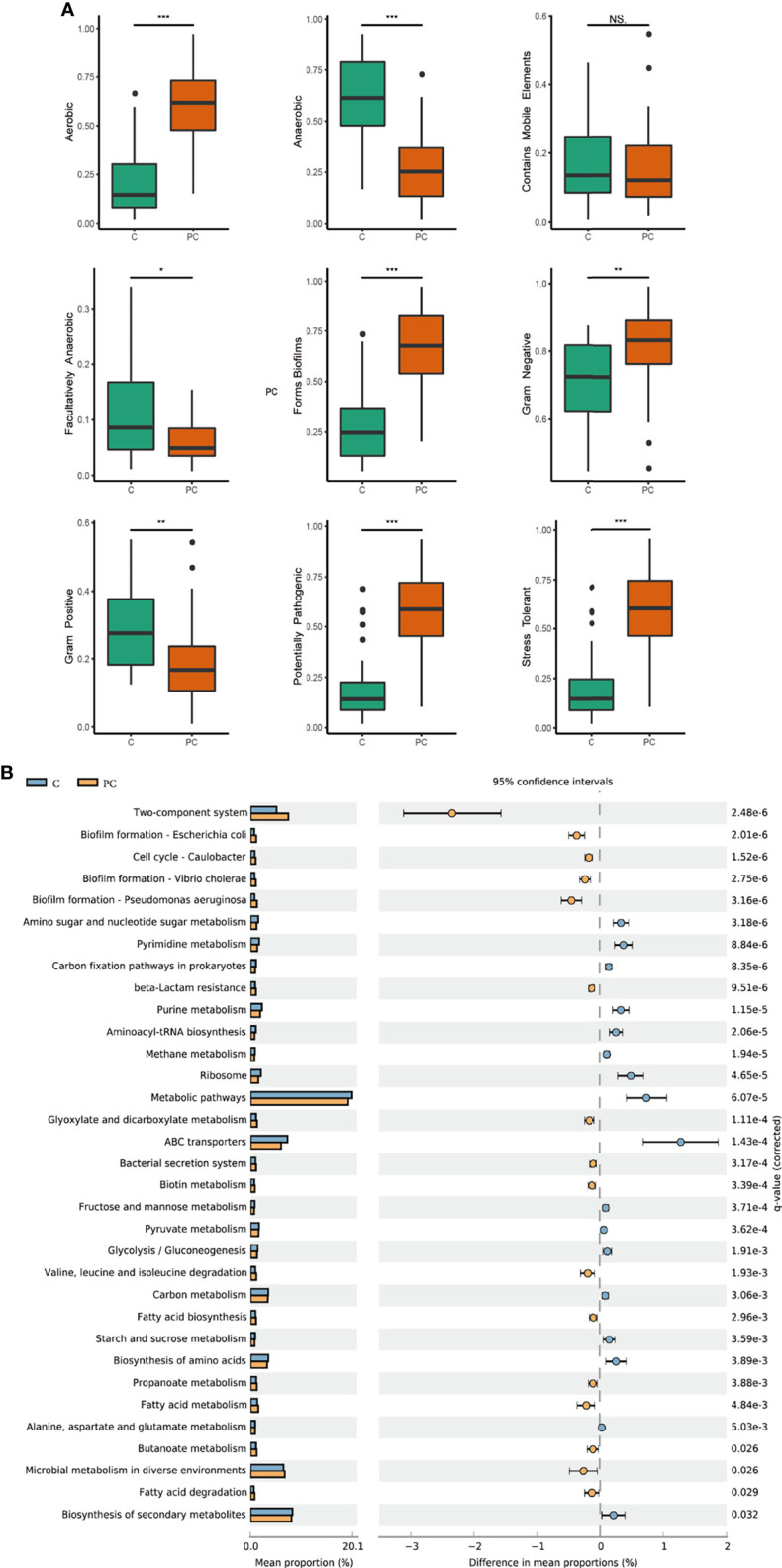
Prediction of microbiome phenotypes and functions. **(A)** Phenotypes prediction was conducted by BugBase analysis, including gram-positive, gram-negative, biofilm forming, stress tolerance, potential pathogenicity, aerobic, anaerobic, facultatively aerobic and mobile elements. **(B)** Microbial functional profile was predicted by Tax4Fun2 based on the KEGG pathway, and statistically analyzed by STAMP. KEGG pathways with significant abundance difference (P < 0.05) are shown. C, tumor tissue; PC, paracancerous tissue. ns, no significance; *P < 0.05, **P < 0.005, ***P < 0.001.

BugBase was used to predict and compare the microbial phenotype across the six sample locations. As shown in **Supplementary Figure 4**, tumor tissue was characterized by the highest abundance of anaerobes, while the highest abundance of aerobic, gram-negative bacteria, biofilm formation, potential pathogenicity, and stress tolerance were found in the paracancerous tissue. Tumor and paracancerous tissues both showed a low abundance of mobile elements. Upon comparing tumor tissue and paracancerous tissue, tumor tissue exhibited a greater abundance of gram-positive bacteria, anaerobes, and facultative anaerobes than paracancerous tissue, while gram-negative bacteria, aerobic, biofilm formation, potential pathogenicity, and stress tolerance were more frequent in the paracancerous tissue (all p < 0.05; **Figure 6A**).

Tax4Fun2 analysis was used to predict and compare changes in microbial function and metabolism pathways between the two groups (**Figure 6B**). Functions related to nucleotide metabolism, including amino sugar and nucleotide sugar metabolism, purine, and pyrimidine metabolism, as well as functions related to mRNA translation including ribosome and aminoacyl tRNA biosynthesis, were significantly enriched in tumor tissue (P < 0.05). Several metabolic pathways related to biosynthesis, energy supply, such as biosynthesis of amino acids, carbon fixation pathways, carbon metabolism, fructose and mannose metabolism, pyruvate metabolism, glycolysis/glucogenesis, and ABC transporters were also more abundant in tumor tissue (P < 0.05), While biofilm formation, fatty acid biosynthesis, metabolism and degradation were decreased.

## Discussion

The oral microbiome plays an essential role both in the stability and balance of oral microecology and host defense. Once homeostasis is disturbed, an imbalance of microbial flora contributes to oral diseases such as dental caries, periodontitis, and oral mucosal diseases, and systemic diseases, such as cardiovascular disease, diabetes, rheumatoid arthritis, Alzheimer’s disease, and head and neck cancers (Sampaio-Maia et al., 2016; Long et al., 2017; Kilian, 2018; Sudhakara et al., 2018; Sureda et al., 2020; Radaic and Kapila, 2021). There is much evidence that the colonization, translocation, and imbalance of oral microflora play key roles in OSCC, providing potential biomarkers for the occurrence, development, and prognosis of OSCC (Wang and Ganly, 2014; Sun et al., 2020). However, at present, differences in oral microbiome across multiple oral niches in OSCC patients have not been truly investigated.

In the present study, samples were collected from 65 patients with OSCC, and 16S rDNA sequencing was used to characterize the microbial profile in tumor tissue, paracancerous tissue, cancer surface, contralateral normal mucosa, saliva, and tongue coat to evaluate associations between the oral microbiome and OSCC. Significant differences in microbial composition and function were found between the six different oral niches. The diversity and uniformity of tumor tissue were found to be higher than that of other niches, as indicated by the Shannon and Simpson diversity indices, consistent with previous findings (Zhao et al., 2017; Yang et al., 2018; Su et al., 2021). However, some contradictory results have also been reported, such as decreases in the diversity of bacterial communities in tumor samples (Pushalkar et al., 2012; Schmidt et al., 2014; Wang et al., 2017; Sarkar et al., 2021).

Previous studies have shown *Proteobacteria*, *Firmicutes*, *Bacteroidetes*, *Fusobacteria*, and *Actinobacteria* to be the dominant phyla (Schmidt et al., 2014; Guerrero-Preston et al., 2016; Al-Hebshi et al., 2017b; Wang et al., 2017; Sarkar et al., 2021), in support of our findings. The *Firmicutes* have been reported to be the most abundant phylum in the oral microbiome (Schmidt et al., 2014; Guerrero-Preston et al., 2016; Perera et al., 2018; Sarkar et al., 2021). In this study, *Firmicutes* was the most abundant phylum in contralateral normal mucosa and saliva, and the microflora of the two niches was similar at the phylum, genus, and species levels. Upon comparing tumor tissue and paracancerous tissue, the tumor tissue demonstrated greater populations of *Fusobacterium*, *Prevotella*, *Porphyromonas*, *Campylobacter*, *Aggregatibacter*, *Treponema*, and *Peptostreptococcus*, and lower populations of *Stenotrophomonas*, *Neisseria*, *Sphingomonas*, and *Veillonella*. Most of the genera significantly enriched in tumor tissue samples were those that have been associated with periodontal diseases, in agreement with previous studies (Zhao et al., 2017; Li et al., 2020; Kavarthapu and Gurumoorthy, 2021). Periodontitis-associated bacteria promote the production of pro-inflammatory mediators, such as interleukin, TNF-α, and matrix metalloproteinase, which are released into the oral microenvironment, causing chronic inflammation, and promoting tumor cell migration and invasion (Champagne et al., 2003; Tezal et al., 2009; Schmidt et al., 2014; Li et al., 2020). Fusobacterium, a normal component of the oral microbiome, coexists with other bacteria in dental plaque, forming a bridge between early and late colonizers (Zhao et al., 2017). The virulence factors produced by Fusobacterium, such as adhesins, LPS, and RadD, have been associated with aberrant immune responses, chronic infection, modulating oral carcinogenesis, and promoting cancer progression (Gholizadeh et al., 2017; De Andrade et al., 2019). Aggregatibacter has been associated with invasive periodontitis (Ando et al., 2012) and can induce inflammation through cytolethal distendin toxin (CDT), leukotoxin and lipopolysaccharide (LPS) (Herbert et al., 2016). Further, the abundance of *Neisseria*, *Veillonella*, *Rothia*, and *Streptococcus* had been reported to decrease significantly in tumor tissue, which may be related to a decrease in the health of the oral cavity (Pushalkar et al., 2012; Zhao et al., 2017; Al-Hebshi et al., 2017b; Sun et al., 2020).

At the species level, the relative abundances of *Porphyromonas endodontalis*, *Campylobacter gracilis*, *Peptostreptococcus stomatis*, *Prevotella intermedia*, *Eubacterium vuril subsp schtika*, and *Parvimonas micra* in tumor tissue increased in comparison to those in paracancerous tissue. Notably, these florae are all anaerobes or facultative anaerobes. The phenotype prediction also showed that tumor samples exhibited the highest abundances of anaerobes. Tumor cells live in an environment that is comparatively hypoxic and of a lower pH in comparison to healthy tissue, which may be due to the increased metabolic rate of tumor cells combined with an insufficient local blood supply, causing most of the bacteria that are found thriving in tumor tissue to be anaerobes. *Porphyromonas Endodontalis*, a newly discovered periodontal pathogen, was observed to be highly abundant in cancerous tissue (Tezal et al., 2009; Pérez-Chaparro et al., 2014; Gonçalves et al., 2016). *Parvimonas micra* was also reported to be enriched in OSCC tumor lesions and be associated with tumor stages. (Al-Hebshi et al., 2017a; Yang et al., 2018).

We used SparCC analysis to illustrate the microbial network. In tumor tissues, the key species was Clostridium disporicum. Clostridium disporicum was first isolated from rat intestinal flora in 1987 (Horn, 1987). Otherwise, Clostridia are an important component of the human intestinal anaerobic flora, so the predisposing factors of clostridial infections are commonly associated with malignancy or antibiotherapy (Mallozzi et al., 2010). As a common conditional pathogen, Clostridium can colonize to colonic epithelial cells and produce carcinogenic substances to promote tumorigenesis.

ROC curve analysis is typically used to evaluate the discrimination ability and diagnostic efficacy of a predictive model. In this study, the ROC model containing *Lautropia*, *Asteroleplasma*, *Parvimonas*, *Peptostreptococcus*, *Pyramidobacter, Roseburia*, and *Propionibacterium* provided a good level of prediction accuracy for OSCC metastasis, with a statistically significant diagnostic accuracy of 82.3%, suggestive of a significant correlation with the metastasis of OSCC. OSCC is prone to early transfer to the regional lymph nodes, so the tumor metastasis trend is an important predictor of survival outcomes in OSCC patients. Mager et al. found that high abundance of several bacterials in salivary may be diagnostic indicators of OSCC (Mager et al., 2005). Wei et al. proved that salivary metabolomics (Valine, lactic acid, and phenylalanine) had the potential for detection of OSCC (Wei et al., 2011). Furthermore, two other studies have demonstrated good diagnostic power of oral microbiome for the OSCC (Zhao et al., 2017; Li et al., 2020). The above studies compare OSCC patients to healthy control individuals, while our research performed a comparison between patients with tumor metastasis and patients without lymph nodes and other organ metastases, drawing the conclusion of good diagnostic ability of oral microbiome for OSCC metastases. Our study has some limitations in samples and methodology, further research is needed to confirm the present results.

Finally, Tax4Fun2 was used to predict the functional profile based on 16S rDNA gene sequences, furthering the understanding of the significance of the oral microbiome. Nucleotide metabolism is an important pathway providing purine and pyrimidine molecules for DNA replication and RNA biogenesis (Siddiqui and Ceppi, 2020), and mRNA translation is a critical process of gene expression and protein synthesis. The current study showed that functions related to nucleotide metabolism and mRNA translation, such as amino sugar and nucleotide sugar metabolism, purine and pyrimidine synthesis, ribosome and aminoacyl tRNA biosynthesis, and the functions related to biosynthesis and energy supply were significantly enriched in OSCC samples, likely reflecting the enhanced fundamental requirements for bacterial life in the OSCC habitat and specific adaptation to distinct micro-ecological environment. The variations of bacterial metabolic pathways in tumor tissues indicated a contribution of the oral microbiome to creating the tumor microenvironment through the biosynthesis of secondary metabolites (Zhao et al., 2017; Li et al., 2020).

In summary, the present article reports a comprehensive comparison of the microbiome across different oral niches in patients with OSCC, and these findings reveal differences in the characteristics of the oral microflora. The human oral microbiome has been demonstrated to be site-specific (Tanner et al., 2006), and the present study further elucidates species similarities and differences among microbial communities in different oral niches. Periodontitis-related flora and anaerobes were shown to be significantly enriched in tumor tissue. Further, the microorganisms in tumor tissue might be potential indicators of the development and metastasis of OSCC. This study provides evidence that the dynamic balance between the resident oral microflora and the host is altered in OSCC, which may be the key mechanism by which oral symbiotic bacteria promote or prevent the occurrence of oral cancer. This study had some limitations. Firstly, the taxonomic accuracy of 16S rDNA was limited (Zhao et al., 2017; Johnson et al., 2019). Recent studies have shown that although more than 99% of the sequencing analyses can be correctly classified at the genus level, many bacteria are misclassified (Winand et al., 2019). Further, many bacteria have not yet been sequenced or discovered. Also, the exact pathogenesis of this phenomenon is still to be clarified and it is not currently possible to determine with sufficient certainty, based on these data, whether the observed bacterial changes contribute to the carcinogenesis or progression of OSCC.

## Data Availability Statement

The original contributions presented in the study are publicly available. This data can be found here: [http://www.ncbi.nlm.nih.gov/bioproject/866676/PRJNA866676].The sequencing data generated in this study are submitted to the Sequence Read Archive of the National Center for Biotechnology Information database (accession number: PRJNA866676).

## Ethics Statement

The studies involving human participants were reviewed and approved by Ethics Committee of Qilu Hospital of Shandong University. The patients/participants provided their written informed consent to participate in this study.

## Author Contributions

FN: Conceptualization, Investigation, Writing-original draft. LW: Conceptualization, Investigation, Methodology, Software, Writing-review & editing. YH: Conceptualization, Investigation,Writing-review & editing. PY: Conceptualization, Supervision, Writing-review & editing. PG: Conceptualization, Investigation,Writing-review & editing. QF: Conceptualization, Formal analysis, Investigation, Visualization, Supervision, Writing-review & editing. CY: Formal analysis, Data curation, Supervision, Project administration. All authors contributed to the article and approved the submitted version.

## Funding

This work was supported by the National Natural Science Foundation of China (No. 81702684). National Natural Science Foundation of China (No. 82071122), The Taishan Young Scholars of Shandong Province (tsqn 201909180), Oral Microbiome Innovation Team of Shandong Province (No. 2020KJK001), The National High-level Young Scientist Project Foundation (2019), Excellent Young Scientist Foundation of Shandong Province (No. ZR202102230369) and periodontitis microbiome innovation team of Jinan City (2021GXRC021).

## Conflict of Interest

The authors declare that the research was conducted in the absence of any commercial or financial relationships that could be construed as a potential conflict of interest.

## Publisher’s Note

All claims expressed in this article are solely those of the authors and do not necessarily represent those of their affiliated organizations, or those of the publisher, the editors and the reviewers. Any product that may be evaluated in this article, or claim that may be made by its manufacturer, is not guaranteed or endorsed by the publisher.
